# Using Passive Surveillance to Maintain Elimination as a Public Health Problem for Neglected Tropical Diseases: A Model-Based Exploration

**DOI:** 10.1093/cid/ciae097

**Published:** 2024-04-25

**Authors:** Amanda Minter, Graham F Medley, T Déirdre Hollingsworth

**Affiliations:** Big Data Institute, Li Ka Shing Centre for Health Information and Discovery, University of Oxford; Department of Global Health and Development, London School of Hygiene and Tropical Medicine, United Kingdom; Big Data Institute, Li Ka Shing Centre for Health Information and Discovery, University of Oxford

**Keywords:** neglected tropical diseases, mathematical modelling, elimination

## Abstract

**Background:**

Great progress is being made toward the goal of elimination as a public health problem for neglected tropical diseases such as leprosy, human African trypanosomiasis, Buruli ulcer, and visceral leishmaniasis, which relies on intensified disease management and case finding. However, strategies for maintaining this goal are still under discussion. Passive surveillance is a core pillar of a long-term, sustainable surveillance program.

**Methods:**

We use a generic model of disease transmission with slow epidemic growth rates and cases detected through severe symptoms and passive detection to evaluate under what circumstances passive detection alone can keep transmission under control.

**Results:**

Reducing the period of infectiousness due to decreasing time to treatment has a small effect on reducing transmission. Therefore, to prevent resurgence, passive surveillance needs to be very efficient. For some diseases, the treatment time and level of passive detection needed to prevent resurgence is unlikely to be obtainable.

**Conclusions:**

The success of a passive surveillance program crucially depends on what proportion of cases are detected, how much of their infectious period is reduced, and the underlying reproduction number of the disease. Modeling suggests that relying on passive detection alone is unlikely to be enough to maintain elimination goals.

Huge progress has been made toward achieving the goals laid out in the World Health Organization (WHO) road map for neglected tropical diseases (NTDs), with multiple countries declaring elimination as a public health problem (EPHP) for trachoma, lymphatic filariasis, and other infections. The WHO has defined disease elimination–specific goals in the road map for the next decade, with the EPHP goal defined as reduction in observable prevalence below a defined threshold [1]. The impact of the coronavirus disease 2019 (COVID-19) pandemic and other interruptions to programs mean that the attainment of these goals will be challenging, but momentum continues [2–4]. In addition, post-validation strategies for maintaining these goals are still an open practical and research question [5]. One important pillar of sustaining the goals will be passive detection of cases, particularly for diseases for which case finding is the major method of control, such as leprosy, human African trypanosomiasis (HAT), Buruli ulcer, and visceral leishmaniasis.

Passive surveillance is the routine detection of cases via individuals self-reporting to care at the lowest levels of the healthcare system due to symptoms of disease. Passive surveillance can be used in a responsive surveillance model, where changes in case numbers could trigger contact or household tracing, or mass screenings. However, the thresholds for these triggers are often not well defined.

The impact on maintaining elimination goals if surveillance efforts are reduced when observed cases numbers reach EPHP thresholds could be severe. Following improved (active) surveillance, there may be an apparent increase in cases followed by a reduction when detected cases are treated (Figure 1). Once active surveillance is stopped and surveillance is solely passive, prevalence levels may appear to have reached a low level in the population, when cases are actually increasing over time. Epidemic growth rates for many, but not all, NTDs are relatively slow, and therefore passive case detection could detect resurgence in a timely way, but only if it is effectively implemented, which may be challenging as many of these diseases are not associated with care seeking in endemic populations, or care is only sought for the minority of cases that are severe or late in their progression.

**Figure 1.**
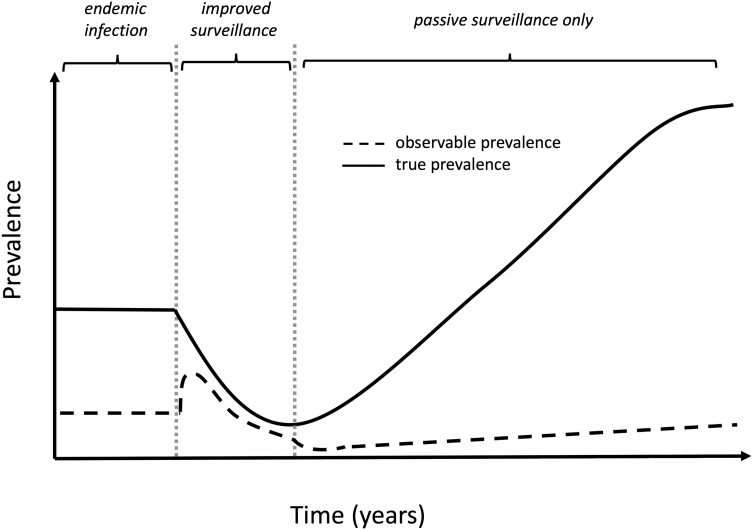
Sketch of the relationship between true prevalence and the observed prevalence from detected cases over time. After improved detection, there is an apparent increase in prevalence. Once levels seem to have decreased, if the next stage of surveillance is passive only, infection numbers are likely to increase but detected cases will increase much more slowly. Adapted from Figure 1 in Coffeng et al.[6]

As programs move closer to their elimination goals, passive surveillance may be used as a post-validation strategy. The capability of passive surveillance to maintain transmission at low levels will depend on the underlying disease dynamics, the level of passive detection, and how much an individual's transmissibility is reduced after detection. These will depend on population knowledge of the condition, behavior of patients, and the local population and availability of health facilities and cost/distance to travel. As EPHP validation plans are made, the long-term capability of passive surveillance to detect resurgence must be explored. Here we investigate the limits of passive detection alone in preventing resurgence of NTDs in a model-based study.

## METHODS

**Figure 2.**
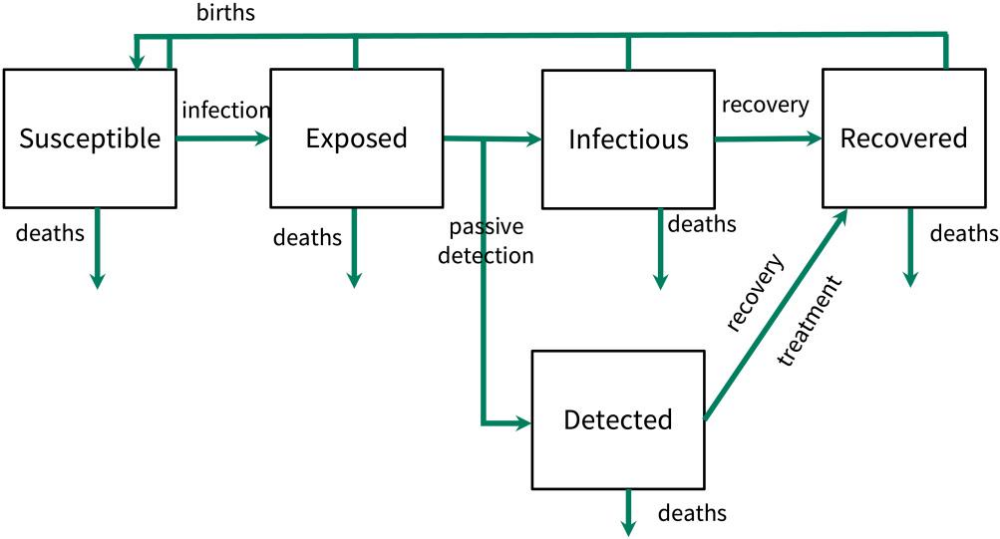
Schematic representation of the model.

To investigate what levels of passive detection are needed to be present to prevent resurgence, we consider a simple model of infection transmission (Figure 2). We formulated a mathematical model to predict the effect of passive detection on preventing epidemics of NTDs (see Supplementary Material - Appendix 1 for more details). The model is described by a system of ordinary differential equations. Individuals can be free from and susceptible to infection (*S*), infected but not yet infectious (*E*), infected and infectious (*I*), detected (*D*), or recovered (*R*).

Individuals become infected at a rate *β*, and detected individuals (*D*) are still infectious until they are treated, so contribute to onward infection. After an average period of 1/σ weeks, individuals become infectious. At this point, a proportion of individuals $p_{E}$ move to the detected class via passive detection, that is, self-reporting after symptom onset.

The remaining $\left(1-p_{E}\right)$ individuals move to the *I* class where they can either recover or die from natural causes.

We evaluate the impact of different interventions through the impact on the basic reproduction number, *R_0_*.

### Reproduction Number

The basic reproduction number, *R_0_*, is the average number of infections generated by a single (average) infected individual in a wholly susceptible population. This can also be evaluated when interventions are in place, sometimes called the reproduction number under control (*R_c_*) or, more commonly, the effective reproduction number (*R*). For this scenario we consider that there is no immunity in the population during the postvalidation period.

The *R*_0_ for infection dynamics only (no detection or treatment) is given by the probability that an individual survives to the infectious period, the transmission rate, and the average duration of infectiousness (see Supplementary Material - Appendix 2):


$$R_{0}=\frac{\sigma }{\left(\mu +\sigma \right)}\beta \frac{1}{\left(\gamma +\mu \right)}.$$

When passive detection and treatment are present, a proportion of individuals are detected and then treated. The expression of the effective reproduction number for this model is


$$R=(1-p_{E})\frac{\sigma }{\left(\mu +\sigma \right)}\beta \frac{1}{\left(\gamma +\mu \right)}+p_{E}\frac{\sigma }{\left(\mu +\sigma \right)}\beta \frac{1}{\left(\gamma +\mu +\rho \right)}.$$

The first part of the expression represents an individual who is not passively detected, multiplied by the probability that they move from exposed to infectious before dying. The number of secondary infections they will contribute depends on the transmission rate while infectious, multiplied by the duration of infectiousness while in the infectious class.

The second part of the expression represents an individual who is passively detected, multiplied by the probability that they move from exposed to infectious before dying. The number of secondary infections they will contribute depends on the transmission rate while infectious, multiplied by the duration of infectiousness while in the detected class, which is usually determined by the delay between diagnosis and treatment.

### Improve Detection or Improve Time to Treatment?

To investigate the benefits of detecting more infections versus treating people quicker once they are detected, we consider the effect of changing these parameter values on the *R.* We define the 2 parts of the *R* expression as undetected transmissions $(1-p_{E})\frac{\sigma }{\left(\mu +\sigma \right)}\beta \frac{1}{\left(\gamma +\mu \right)}$ and detected transmissions $p_{E}\frac{\sigma }{\left(\mu +\sigma \right)}\beta \frac{1}{\left(\gamma +\mu +\rho \right)}$.

The value of the detected and undetected parts of the *R* expression were calculated for different proportions of new infections detected, that is, the proportion of infections that are detected when infectious and for different reductions in the duration of infectiousness once detected (achieved by increasing treatment rate; see Supplementary Material - Appendix 2).

We consider 2 example diseases; one disease with an underlying (no detection or treatment) $R_{0}$ close to the threshold of 1 ($R_{0}=1.09$), and a second with a higher $R_{0}$ ($R_{0}=2.06$) representing a disease dynamic similar to leprosy ($R_{0}$ range, 1.74–2.36; unpublished data). In both cases we assume the same transmission rate but a different recovery rate of 1/3.5 years and 1/7 years, respectively, (leprosy type parameters adapted from [7]).

## RESULTS

**Figure 3.**
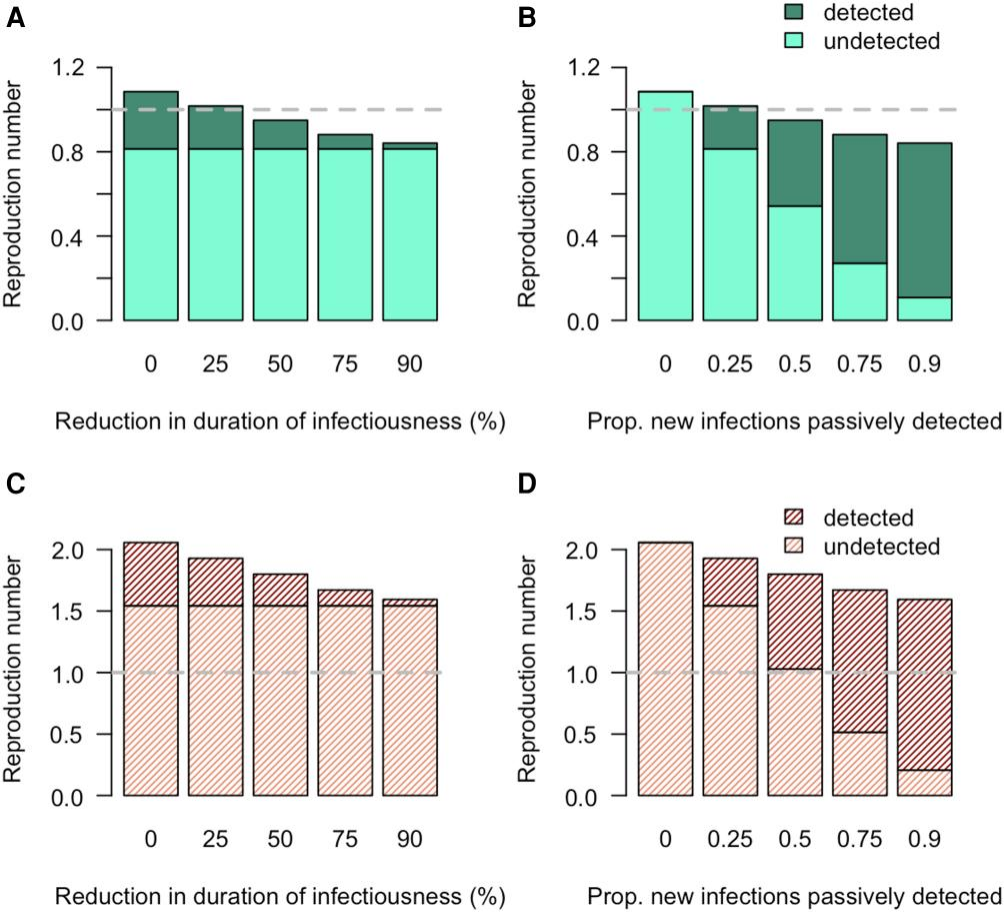
The values of the number of onward transmissions, the effective reproduction number (*R*), due to detected (upper shaded region) and undetected (lower lighter regions) portions of the infectious period for low *R*_0_ (*A* and *B*) and high *R*_0_ (*C* and *D*).

When a fixed proportion of new infections is detected ($p_{E}$ = 0.25), reducing onward transmission of infected cases by reducing their duration of infectiousness (eg, by a curative treatment or by one which halts onward transmission) in the model by increasing the recovery rate due to treatment reduces the average number of onward transmissions once detected (the shaded part of each bar in the left column). Note that when the baseline $R_{0}$ is low (Figure 3*A* and 3*B* ), reducing the duration of infectiousness can reduce *R* to <1. When *R*_0_ is higher (Figure 3*C* and 3*D* ), reducing duration of infectiousness will not reduce *R*_0_ to <1 for the given proportion of new infections detected. Note that this is an average treatment recovery rate and will include treatment failures. This effect on reducing *R* to less than the threshold is limited by the underlying *R*_0_ and, in particular, the infectiousness and the duration of infectiousness for those who are not detected through passive surveillance prior to passive detection (eg, asymptomatic transmission, or transmission from those with poor access to healthcare).

Similarly, with a fixed reduction of the duration of infectiousness of 25%, the effect of increasing the proportion of new infections passively detected in reducing the overall *R* is limited by how frequently detected individuals are treated. Increasing the proportion of new infections passively detected needs to be coupled with an appropriate time to treatment to reduce *R* to <1 (Figure 3*C* and 3*D*, the size of the shaded parts).

## DISCUSSION

The WHO 2030 NTD road map lays out elimination and eradication goals for the next 10 years [1]. The achievability of these goals for case finding diseases will depend on whether a surveillance system can detect resurgence of cases and treat individuals quickly. The ability of passive detection to maintain low levels of transmission, or indeed support the achievement of eradication, will depend on country-specific factors and history of transmission and control [8].

Although we have used a simple model to investigate these effects, our results suggest a general insight that for many NTDs a high proportion of new infections would need to be detected to prevent resurgence. The impact of COVID-19 on NTD programs and health systems more generally has already shown the potential for dramatic drops in diagnosis if active programs are not maintained [2]. A dramatic drop in leprosy diagnoses was observed in 2020 in Brazil (one of the highest-burden countries for leprosy) [3], with the undetected cases continuing to transmit and impact the long-term patterns of incidence.

Passive detection could be improved in a number of ways, but there is a risk that over the decades-long timescale of NTD resurgence, the quality of passive surveillance may weaken as familiarity of the disease symptoms may decrease. An ongoing feature of many of these diseases is multiple visits to health centers for diagnosis, extending their time to diagnosis and duration of infectiousness, as historically observed for *rhodesiense* HAT [9]. Analysis by WHO staff of the situation in South Sudan suggested that challenges in case detection would increase the risk of a resurgence in HAT cases, which stimulated strengthening of the health system [10]. More recent data for *gambiense* HAT in Chad highlights reductions in the rate and time to diagnosis given advances in many aspects of diagnosis and treatment [11]. However, for some diseases (such as visceral leishmaniasis), given the ongoing challenges in diagnostic, there may still be important opportunities to use even imperfect diagnostics to reduce resurgence [12].

Transmission from detected cases also needs to be rapidly halted, through effective treatments or other transmission reducing interventions, to prevent resurgence. Given the long timescale of NTD resurgence, it is hoped that treatment times will be reduced through new drug developments. Reactive policies, which would increase detection and treatment, would strengthen the use of passive surveillance but may be challenging if passive surveillance systems are not able to detect ongoing transmission at low levels [13]. For example, for visceral leishmaniasis prevalence has been shown to have an effect on the effectiveness of passive and active case detection [14]. These types of dynamics could be due to both program effects (how good healthcare is at diagnosing when a case turns up) and population effects (how good people are at going to healthcare depending on awareness and perception of risk).

Our analysis separates probability of detection and duration of infectiousness of those cases that are detected as independent effects, whereas in terms of both creating the demand (eg, sensitizing the population) and improving access (eg, building more primary care centers, or providing more diagnosis closer to the patient) these 2 things will be correlated—the more cases that are detected, the earlier you find them, and so in some populations there will be a jointly positive effect, whereas in others there will be less detection and less impact of being detected on an individual's infectiousness, due to late detection or poor treatment. Depending on the disease being considered, and the characteristics of those who are more likely to be detected, it may be better to detect 75% of the patients and reduce their onward transmission by 25%, whereas for other diseases it might be more important to detect 100% of the patients and reduce their onward transmission by 50%

Our model-based study included several other simplifications, such as excluding passive detection via other routes, for example, healthcare visits for other purposes. We also did not include loss of immunity or preexisting immunity in the population. Including these extensions may have reduced the magnitude of the results, but the overall conclusions would be similar.

To validate elimination goals, there is a need to understand the disease-specific indicators of resurgence [15]. Given that detection, surveillance, and treatment parameters are very challenging to measure, surrogate measures that indirectly inform about underlying transmission will be needed—for example, identifying changes in age at diagnosis, onset of symptoms to diagnosis, or extent of symptoms at diagnosis. Modeling has a role to play in evaluating these measures, the likelihood programs have reached their elimination goals [16], exploring the effect of different levels of passive surveillance on elimination [17], and if additional active surveillance or even vector surveillance is required [18].

Our results highlight many already-known limits to passive detection and surveillance. To ensure the elimination goals are maintained, a robust, responsive surveillance system will be needed. There needs to be long-term investment in strengthening health systems and in support of the universal healthcare movement, but these investments will be particularly challenging in a time of proposed cuts to NTD programs.

## Supplementary Data

Supplementary materials are available at *Clinical Infectious Diseases* online. Consisting of data provided by the authors to benefit the reader, the posted materials are not copyedited and are the sole responsibility of the authors, so questions or comments should be addressed to the corresponding author.

## Supplementary Material

ciae097_Supplementary_Data

## Notes

***Author contributions.*** A. M. and T. D. H. conceived and designed the study. A. M. designed and implemented the analysis and wrote the first draft. G. F. M. commented on the analysis and edited the manuscript.

***Data availability.*** The code to reproduce all these model outputs is available for reuse.

***Financial support.*** The authors are grateful for funding by the Bill & Melinda Gates Foundation via the NTD Modelling Consortium (grant number INV-030046).

***Supplement sponsorship.*** This article appears as part of the supplement “New Tools and Nuanced Interventions to Accelerate Achievement of 2030 Roadmap for Neglected Tropical Diseases,” sponsored by funding of Professor T. Déirdre Hollingsworth's research by the Li Ka Shing Foundation at the Big Data Institute, Li Ka Shing Centre for Health Information and Discovery, University of Oxford; and funding of the NTD Modelling Consortium by the Bill & Melinda Gates Foundation (INV-030046).
