# Supplementary material for: Using Passive Surveillance to Maintain Elimination as a Public Health Problem for Neglected Tropical Diseases: A Model-Based Exploration
Source: Clin Infect Dis. 2024 Apr 25;78(Suppl 2):S169–74. doi: 10.1093/cid/ciae097 (PMC11088853; doi:10.1093/cid/ciae097)
Supplement: ciae097_Supplementary_Data [file ciae097_supplementary_data.docx]

## Supplementary Material

Using Passive Surveillance to Maintain Elimination as a Public Health Problem for Neglected Tropical Diseases - A Model-Based Exploration

Amanda Minter, Big Data Institute, Li Ka Shing Centre for Health Information and Discovery, University of Oxford, UK*

Graham F. Medley, Department of Global Health and Development, London School of Hygiene and Tropical Medicine, London, UK

T. Déirdre Hollingsworth, Big Data Institute, Li Ka Shing Centre for Health Information and Discovery, University of Oxford, UK

*Corresponding author

## Appendix 1 : Model detail

We formulated a mathematical model to predict the effect of passive surveillance on preventing epidemics of NTDs (see main text for model schematic). The model is described by a system of ordinary differential equations. Individuals can be free from and susceptible to infection ($S$), infected but not yet infectious ($E$), infected and infectious ($I$), detected ($D$) or recovered ($R$).


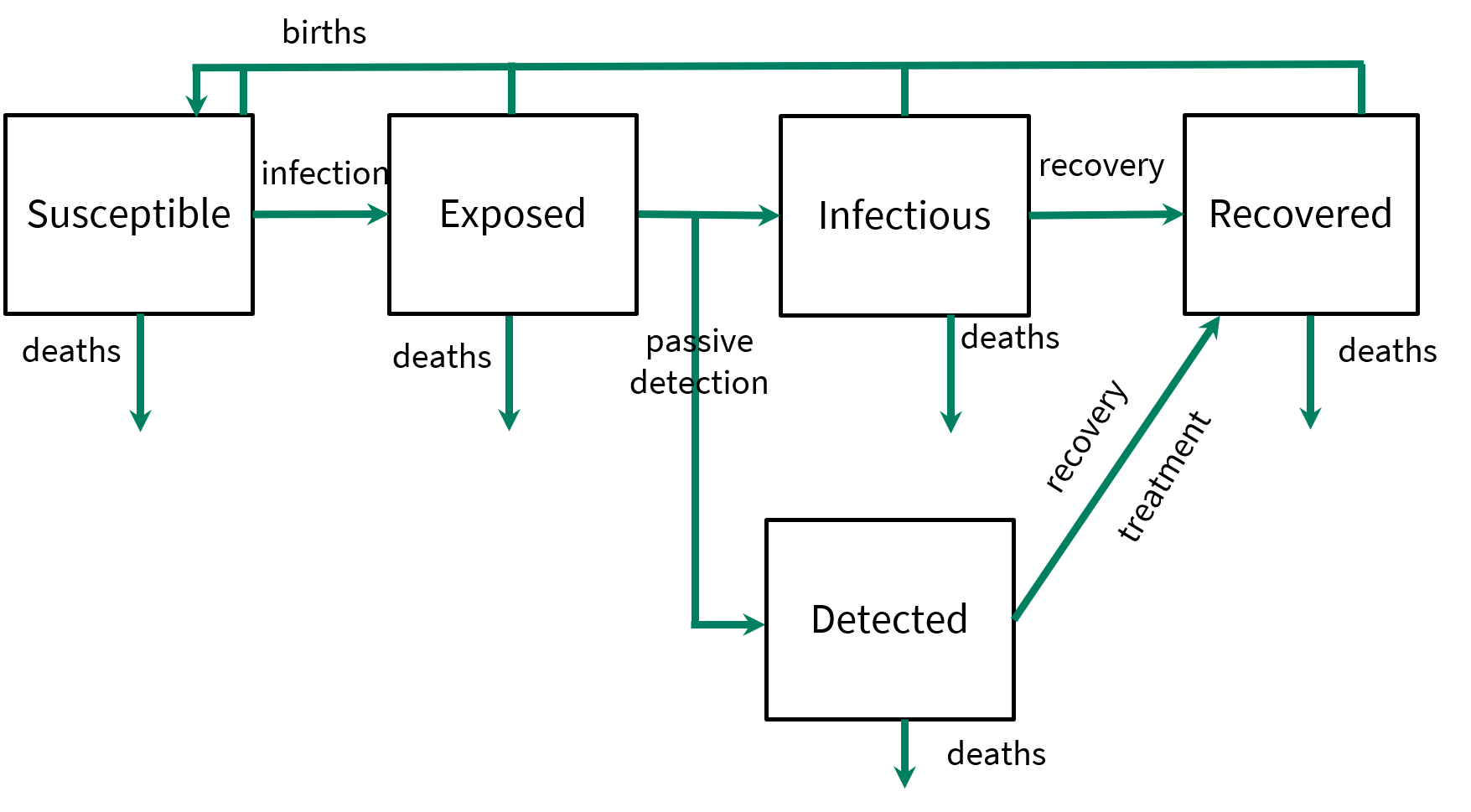


Individuals become infected at a rate $\beta$, detected individuals ($D$) are still infectious until they are treated, and so contribute to onward infection. After an average period of $1/\sigma$ weeks, individuals become infectious. At this point, a proportion of individuals $p_{E}$ moves to the detected class via passive surveillance i.e. self-reporting after symptom onset.

The remaining $(1-p_{E})$ individuals move to the $I$ class where they can either recover or die from natural causes.

While in the detected class, individuals are still infected and infectious, therefore they can recover from infection and contribute to further infections. Individuals in the detected class are treated at rate $\rho$, once treated, they move to the recovered class.

$$\begin{matrix} \frac{dS}{dt} & =\mu N-\frac{\beta S(I+D)}{N}-\mu S \\ \frac{dE}{dt} & =\frac{\beta S(I+D)}{N}-(\sigma+\mu)E \\ \frac{dI}{dt} & =(1-p_{E})\sigma E-(\gamma+\mu)I \\ \frac{dR}{dt} & =\gamma I+\rho D-\mu R \\ \frac{dD}{dt} & =p_{E}\sigma E-(\mu+\gamma+\rho)D \end{matrix}$$

In addition, we add compartments to track the number of treated cases ($C$)and the number of untreated cases ($U$), defined as those people who recover or die from natural causes before being treated.

$$\begin{matrix} \frac{dC}{dt} & =\rho D \\ \frac{dU}{dt} & =\gamma(I+D)+\mu(E+I+D) \end{matrix}$$

When a single infectious case is introduced to a susceptible population, the observable prevalence (detected class only) is much smaller than the true prevalence when only passive surveillance is present ($p_{E}=0.01$). The number of untreated cases (those individuals that recover before being treated), is also much higher than the number of treated cases. The number of treated individuals over this timescale would not provide an informative indicator of the underlying infection numbers.


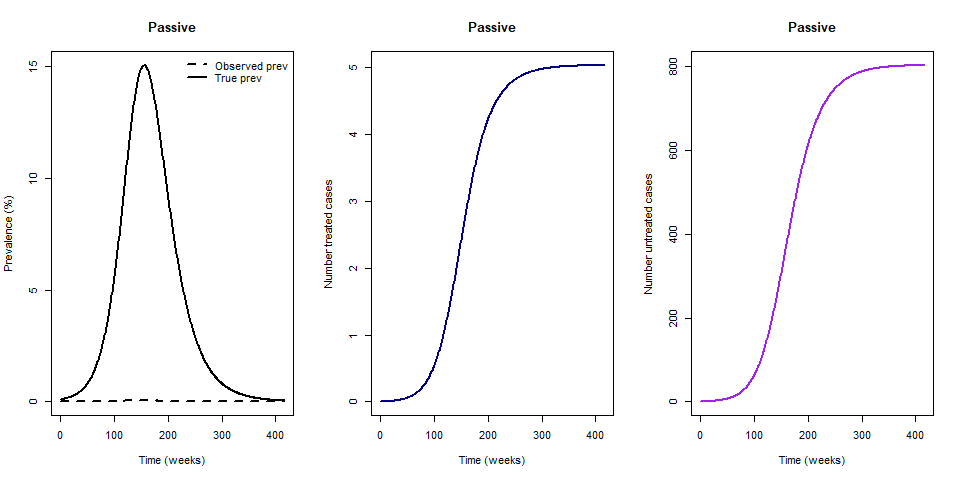


# Appendix 2 : The basic reproduction number, R0

We use the next generation matrix method to find the analytical expression for the basic reproduction number $R_{0}$. We start by identifying the infection terms and removals from the classes $E$, $I$ and $D$.

The new infection terms in $E$,

$$F_{E}=\frac{\beta S(I+D)}{N}$$

and all other removals from $E$ are,

$$V_{E}=(\sigma+\mu)E.$$

The new infection terms in $I$,

$$F_{I}=0$$

and all other removals from $I$ are,

$$V_{I}=(\gamma+\mu)I-(1-p_{E})\sigma E.$$

The new infection terms in $D$,

$$F_{D}=0$$

and all other removals from $D$ are,

$$V_{D}=(\rho+\gamma+\mu)D-p_{E}\sigma E.$$

The $F$ matrix evaluated at the disease free equilibrium is,

$$F=\left[ \begin{matrix} 0 & \beta& \beta\\ 0 & 0 & 0 \\ 0 & 0 & 0 \end{matrix} \right]$$

and the $V$ matrix is,

$$V=\left[ \begin{matrix} (\sigma+\mu) & 0 & 0 \\ -(1-p_{E})\sigma& (\gamma+\mu) & 0 \\ -p_{E}\sigma& 0 & (\rho+\gamma+\mu) \end{matrix} \right].$$

$V^{-1}$ is,

$$V^{-1}=\left[ \begin{matrix} \frac{1}{(\mu+\sigma)} & 0 & 0 \\ \frac{(1-p_{E})\sigma}{(\mu+\sigma)(\gamma+\mu)} & \frac{1}{(\gamma+\mu)} & 0 \\ \frac{\sigma(p_{E}(\gamma+\mu))}{(\mu+\sigma)(\gamma+\mu)(\gamma+\mu+\rho)} & 0 & \frac{1}{(\gamma+\mu+\rho)} \end{matrix} \right].$$

The spectral radius of the matrix $FV^{-1}$ is,

$$R_{0}=(1-p_{E})\frac{\sigma}{(\mu+\sigma)}\beta(\frac{1}{(\gamma+\mu+\theta)})+p_{E}\frac{\sigma}{(\mu+\sigma)}\beta\frac{1}{(\gamma+\mu+\rho)}.$$

The first part of the expression represents an individual who is not passively detected ($1-p_{E}$), multiplied by the probability that they move from $E$ to $I$ before dying ($\frac{\sigma}{(\mu+\sigma)}$). The number of secondary infections they will contribute depends on the transmission rate while infectious ($\beta$) multiplied by the duration of infectiousness while in the $I$ class ($\frac{1}{(\gamma+\mu)}$).

The second part of the expression represents an individual who is passively detected $p_{E}$ multiplied by the probability that they move from $E$ to $I$ before dying ($\frac{\sigma}{(\mu+\sigma)}$). The number of secondary infections they will contribute depends on the transmission rate while infectious ($\beta$) multiplied by the duration of infectious while in the $D$ class ($\frac{1}{(\gamma+\mu+\rho)}$).

# Appendix 3 : Relative reduction in duration of infectiousness in the detected class, $\boldsymbol{r}_{\boldsymbol{dur}}$

The relative reduction in duration of infectiousness in the detected class, $r_{dur}$ is defined as:

$$\begin{matrix} r_{dur} & =1-\frac{\frac{1}{\gamma+\mu+\rho}}{\frac{1}{\gamma+\mu}} \\ & =1-\frac{\gamma+\mu}{\gamma+\mu+\rho} \end{matrix}$$

We can also specify this relationship as a function of treatment rate ($\rho$):

$$\rho=\frac{r_{dur}}{1-r_{dur}}(\gamma+\mu)$$

where $r_{dur}\in(0,1)$.
